# Potential efficacy of parent-infant psychotherapy with mothers and their infants from a high-risk population: a randomized controlled pilot trial

**DOI:** 10.1186/s40814-021-00946-5

**Published:** 2021-11-24

**Authors:** J. Mattheß, M. Eckert, O. Becker, C. Ludwig-Körner, L. Kuchinke

**Affiliations:** International Psychoanalytic University, Stromstr. 3b, 10555 Berlin, Germany

**Keywords:** Young motherhood, Maternal mental health problems, Parent-infant psychotherapy, Attachment, High-risk population, Maternal sensitivity, Mother-Child Facilities

## Abstract

**Background:**

Psychotherapy of mother-child dyads is an intervention which was developed to prevent maltreatment and negative children’s development. There is a lack of good-quality research investigating psychotherapeutic interventions and social care for mothers at high-risk living in Mother-Child Facilities in Germany. The present randomized controlled pilot trial (RCT) aimed to evaluate the need for parent-infant psychotherapy (PIP) and to explore its impact on the mother-infant relationship. Primary feasibility objectives were recruitment and attrition, with potential efficacy defined as the secondary feasibility objective.

**Methods:**

This pilot RCT focused on (young) mothers with cumulative risk factors and their infants under 7 months of age living in Mother-Child Facilities. *N*=32 mother-child dyads were randomly allocated to PIP or Care as usual (CAU). Outcomes were assessed at baseline, 3 months, and 6 months of intervention. The primary potential efficacy outcome was maternal sensitivity. Secondary outcomes were maternal mental health problems, reflective functioning, parenting stress, personality organization, infant’s development, and attachment.

**Results:**

At baseline, all mothers showed low levels of emotional availability, but results revealed improvements in sensitivity, mental health problems, stress, and depressive symptomatology favoring PIP after 6 months. Positive developments in maternal sensitivity, a healthy aspect of mother-child interaction, were only found in the PIP group. Overall attrition was high at 6 months. Some evidence of fewer depressive symptoms and lower maternal distress after 6 months of PIP-intervention exists that did not reach significance.

**Conclusion:**

Findings revealed improvements in the mother’s well-being for both groups, but PIP had a higher impact on the mother-child dyad. In sum, there is some evidence that PIP may represent an effective intervention offer besides the social and pedagogical support in these facilities, but further research is demanded.

**Trial registration:**

DRKS00022485 (retrospectively registered).

## Key messages regarding feasibility

- What areas of uncertainty regarding feasibility existed prior to this study?

There were uncertainties regarding the barriers to participant recruitment and retention in mothers and their children living in Mother-Child Facilities. Further, there was a lack of high-quality research in this area so that there was a need for piloting to identify potential strategies for improving recruitment and retention and to test the research methods.

- What are the key feasibility findings?

Key feasibility findings were a lack of motivation and interest to participate in the study. In addition, most of the participating mothers were concerned about study goals and reported discomfort about psychotherapeutic interventions, as well as negative experiences with the healthcare system. There were not enough incentives for remaining in the study or taking part in it in the first place.

- What are the implications of the feasibility findings for the design of the main study?

The implications for the design of the larger RCT are to consider problems with recruitment and retention. For this purpose, strategies for improving time scheduling like fitting into daily routine of the mother-child dyads and verbal reminders may be beneficial. Emphasis should be given on the motivational side of participation by providing more information on the positive effects such interventions potentially have for the mothers and their children. Retention might be further increased by the availability of home visits in case a mother-child dyad leaves the facility. Future studies need also to consider a potential effect of social and pedagogical care which should be addressed in power analyses and sample size calculation. Before conducting a RCT qualitative studies would be beneficial as well.

## Introduction

Maternal risk factors like early motherhood, mental health problems, or trauma can lead to a lack of maternal sensitivity and reflective functioning up to a situation where mothers are unable to support the child sufficiently in coping with early developmental and maturing issues. In high-risk populations, the cumulation of these factors bears a higher possibility of child’s adverse development and maltreatment. In contrast, a stable parent-child relationship is seen as an essential resilience factor for a healthy mental, physical, and secure attachment development of the child [1–5]. The present feasibility study examines the potential efficacy of a dyadic parent-infant psychotherapy (PIP) with (young) mothers at risk and their children with an age up to 7 months living in Mother-Child Facilities.

Early motherhood is seen as a risk for the development and well-being of the child. Especially adolescent mothers aged between 15 and 21 are considered of being at higher risk for adverse developments [1, 6]. In the transition from adolescence to adulthood, giving birth increases the exposure of child maltreatment, neglect, and adverse child development as well as the risk of maternal mental health problems [7–10]. Particularly young mothers suffer from depressive mood and future anxiety which may interfere with sensitive and emotionally available parenting. The cumulation of further risk factors like absence of partner, poverty, own history of childhood trauma, and high level of psychological stress makes these mothers vulnerable for impaired maternal functioning in the postpartum period and may influence sensitivity towards the infant [6, 8, 10, 11]. (Young) mothers with cumulative risk factors are therefore subsumed as a population at high-risk and are likely to be under the persistent supervision of the German youth welfare. According to recent reviews, maternal mental health problems, stress, and maltreatment can lead to externalizing and internalizing behavior problems in the child with an increasing risk for transmission of these malignant patterns [11, 12].

Predominantly adolescent and young mothers show less sensitive and more intrusive behavior towards their infants [6, 7, 11, 13, 14]. Studies have shown that in the interaction with the child young mothers in comparison to adult mothers tend to over- and understimulation with less structuring behavior and more misinterpretations of the infant’s signals [6]. This causes several negative developmental effects for the child like behaving in an insecure, passive, or even fearful manner and can lead to behavior problems [15] and social-emotional and cognitive impairments [11, 16]. Nevertheless, children of adult mothers suffering from postpartal mental health problems show a similar vulnerability for adverse development with an increasing risk of insecure attachment development and of clinical emotional and behavioral problems [16–19]. With prevalence rates of up to 21% for postpartal depression, anxiety and obsessive-compulsive disorders and a high comorbidity of these, maternal mental health problems can affect the relationship to the child and impedes the establishment of a secure attachment development [20–22]. In contrast, children with an emotional available and sensitive caregiver are shown to develop an organized attachment and socioemotional regulation throughout childhood.

Even before these women at high-risk become pregnant, most of them fail to have a regular daily routine and have problems coping with everyday life and given up schooling or working [7, 23]. The child is therefore also at higher risk for maltreatment, neglect, or abuse. To ensure a healthy development for the mother-child dyad, mothers with cumulative risk factors receive support in Mother-Child Facilities by the German youth welfare as defined in the German Social Security Code (SGB VIII §19). Mother-Child Facilities offer preventive pedagogical and social support to mothers, fathers, and children aged up to 6 years. Parents in these facilities often display various disadvantages such as mental health problems, very early motherhood, or experienced trauma. In such Mother-Child Facilities live around 8–10 mother-child dyads permanently. They are offered 24/7 social and pedagogical care, being supported in raising and establishing a positive relationship with their child. To facilitate a positive dyadic development, this kind of preventive social support is considered a positive factor for future child development and its life-course trajectories. It can also support mothers coping with their everyday life and occurring feelings of overstraining. The aim of Mother-Child Facilities is to strengthen parenting skills and competences, parent-child attachment, to clarify the common perspective and, if necessary, to separate parent and child [24].

Although most of the mothers living in such facilities are adolescent or young, some of them are also adults. These mothers are not always positive about living in Mother-Child Facilities. Although there is often an obvious need for psychotherapeutic support, many of these institutions do not provide any psychotherapeutic support. With its dyadic approach, parent-infant psychotherapy (PIP) aims to improve the mother-child relationship, the infant’s attachment, and the maternal sensitivity [17, 25–27]. The available evidence indicates that PIP is more effective in subgroups of at-risk populations and specifically for mothers experiencing social adversity or problems [19, 28–30]. Nevertheless, none of these studies have targeted the special risk group of mothers living in Mother-Child Facilities with persistent supervision of the youth welfare. In comparison to other interventions such as parenting programs, family support, and medication [2, 31, 32], PIP shows high effect sizes on attachment development (see [33, 34]). In contrast, intervention programs, promoting positive parenting for young parents, show up mixed results [1]. Bartlett and Easterbrooks [35] discuss a moderating effect of young motherhood for the child’s outcome and reveal that the transmission of neglect is attenuated by mothers experiencing social support. Still, the meta-analysis by Taubner et al. [32] pointed to the fact that many of the early intervention programs are too low in frequency to cover the need for social and therapeutic support and to address the significant risk of child welfare vulnerability in these families. Although prevention programs were more effective in high-risk groups, they show generally smaller effects than psychotherapeutic interventions.

Up till now, many intervention programs in Germany have been developed, mainly without any scientific evaluation [32]. Especially when it comes to Mother-Child Facilities, there is a lack of systematic empirical research [36, 37]. For limiting the influences of negative impairments on the child, research about the effectiveness of such social and pedagogical care and the effectiveness of PIP in this high-risk population is needed. Given the impact of cumulative risk factors in early motherhood and of maternal mental health problems, stress, and trauma on the development of the child, it is important to prevent malignant trajectories of these high-risk mothers and to strengthen the positive development of their children. Besides the pedagogical and social care, there could be a need for therapeutic interventions like PIP that focus explicitly on the mother-child dyad with mental health problems. The aim of this randomized controlled pilot trial was to evaluate the need and the potential efficacy of PIP for mothers at risk and their children living in Mother-Child Facilities. Primary feasibility objectives were recruitment and attrition, while the evaluation of potential efficacy was the main secondary feasibility objective.

## Method

### Design

The study was designed as a monocentric, two-arm, open, randomized controlled pilot trial with parallel groups and 6-month intervention. To minimize selection bias, allocation concealment was set before first data collection. Data were collected at three measurement points: first at the beginning of the intervention (T1), after 3 months (T2), and at the end of the 6-month intervention (T3). This pilot RCT aimed to explore the potential efficacy of PIP (in addition to care as usual) for mothers with cumulative risk factors and their infants up to an age of 7 months from a high-risk population in comparison to care as usual. The study was conducted in Mother-Child Facilities in Berlin, Germany, in which the participants live and receive 24/7 care. The primary outcome of the pilot RCT was maternal sensitivity measured by videotaped dyadic play interactions. Secondary outcomes were maternal mental health problems, reflective functioning, personality development, and maternal stress, as well as infant’s development and infant’s attachment. It was hypothesized that in comparison to care as usual only, PIP increases maternal sensitivity and infant’s attachment.

### Participants

A total of 32 mother-infant dyads were included in the study and were randomly allocated to the PIP group (*n*=16, age: MD=21.5 years, SD=4.07) or the CAU control group (*n* = 16; age: MD=22.1 years, SD=6.09). At time of recruitment the dyads already lived for 1 to 7 months in the facilities, the average age of these mothers was M=21.82 years (SD=5.04) and their infants were on average MD=3.6 months old (SD=2.23). About half of the participants were adolescent mothers under 21 years of age (see Table 1).

**Table 1:** Sociodemographic data of the study sample (*N*=32)

	PIP, *n*= 16	CAU, *n*=16	Total sample, *N*=32
	M *(SD)*	M *(SD)*	M *(SD)*
	*n* (%)	*n* (%)	*n* (%)
Mother age	21.5 *(4.01)*	22.1 *(6.10)*	21.81 *(5.10)*
Infant age	0.03 *(0.03)*	0.04 *(0.02)*	0.04 *(0.02)*
Born in Germany	15 (93.8)	14 (87.5)	29 (90.6)
Single mother	9 (56.3)	10 (62.5)	19 (59.4)
Not single mother	7 (43.8)	6 (37.5)	13 (40.6)
Marital status			
Single	4 (25.0)	6 (37.5)	10 (31.3)
Partner	12 (75.0)	10 (62.5)	22 (68.8)
Educational level			
Low	6 (37.5)	7 (43.8)	13 (40.6)
Medium	9 (56.3)	8 (50.0)	17 (53.1)
High	1 (6.3)	1 (6.3)	2 (6.3)
Vocational education	1 (6.3)	0 (0.0)	1 (3.1)
No vocational education	15 (93.8)	16 (100.0)	31 (96.9)
Employed	1 (6.3)	1 (6.3)	2 (6.3)
Unemployed	12 (75.0)	13 (81.3)	25 (78.1)
Other	3 (18.8)	2 (12.5)	5 (15.6)
Number of children			
1	8 (50.0)	9 (56.3)	17 (53.1)
2	6 (37.5)	4 (25.0)	10 (31.3)
3	2 (12.5)	1 (6.3)	3 (9.4)
> 4	0 (0.0)	2 (12.5)	2 (6.3)

Participants were included if they lived in Mother-Child Facilities in Germany, were under permanent supervision by the youth welfare, and were exposed at psychosocial risk such as unemployment (78.1%), single mother status (59.4%), adolescent mothers (50.0%), and mental health problems (59.4%) (see Table 1). Participants had to speak German at a sufficient level and the infant’s age had to be 7 months or younger. Mothers with an acute psychosis, suicidal tendencies, or substance abuse were excluded from study participation. For participating, they received monetary compensation (30 €).

### Procedure

After enrollment, informed consent to participate, and randomization, data were collected before (T1), at 3 months (T2), and 6 months (T3) of intervention. Enrollment and data acquisition were conducted by independent researchers staff members responsible for data collection were blind for treatment allocation. The randomization list with a 1:1 block allocation to PIP or CAU was computed by an independent statistician (L.K.) who was not involved in preparation or execution of the trial before the start of the study. Randomization list was computed with R statistical software using a personal computer and the blockrand function (v1.1). Allocation was concealed for study personnel, but participants were not blind for treatment allocation. There were no special criteria for discontinuing or modifying the allocated intervention. All participants received care as usual with its social and pedagogical care, but the PIP group received in addition PIP intervention once weekly over a period of 6 months. In total, they had 20 PIP sessions with each session lasting approximately 50 min. All PIP therapists were trained psychotherapists and certified in the PIP method [25] and received at least quarterly supervisions.

After enrollment, the mothers were interviewed (M.I.N.I) [38] and asked to complete questionnaires at baseline and at 3 and 6 months (for details, see Measures below). At every measurement point, 10-min videos of mother-infant free-play interactions were recorded to subsequently code maternal sensitivity by the Emotional Availability Scale (EAS) [16]. The Strange Situation Procedure (SSP) [39] and the infant’s development (ET-6-6 R) [40] were measured once at 6 months.

### Parent-infant psychotherapy and care-as-usual

*Parent-infant psychotherapy* is a psychotherapeutic intervention in which mother-child dyads are treated together. Individual sessions could also be held together with the father or social worker/custodian. PIP aims at fostering the parent-infant relationship by supporting the parents’ ability to understand and mentalize the child’s affective states and by promoting parental self-reflection and sensitivity to support the parent-infant relationship. PIP targets the parent-infant relationship by observing their interaction and identifying their problems. It mainly focuses on improving the parenting behaviors by working on their internal working models aiming to promote optimal infant development and attachment security. As a mentalization-based psychodynamic approach, PIP works with reframing, an appropriate confrontation within a supportive framework, psychoeducation, the development of action strategies, and video-feedback.

*Care-as-usual* represents the standard social and pedagogical care in the Mother-Child Facilities as provided by the German health system. CAU in these facilities differs in its offers from one another and is heterogeneous. It mainly consists of socio-pedagogical and educational care such as housekeeping, guided play lessons, discussion groups, and the integration of fathers or partners. The CAU group does not receive any psychotherapeutic intervention but after a waiting period of 6 months, psychoeducational care was additionally offered to all CAU group participants.

### Feasibility objectives

Recruitment rate of the planned *N*=40 dyads was measured as rates of consent, and completion rates of self-report surveys evaluated to approximate attrition. A drop-out rate of 20% was expected (see [19]); thus, success of feasibility would be determined if *N*=32 dyads remained in the study until T3. Secondary feasibility objectives were the primary and secondary outcomes of the pilot RCT to provide effect size measures, and sample size was to be used for future power analysis to design a large-scale RCT.

### Measures

A sociodemographic questionnaire was administered to gather information such as maternal and child’s age, child gender, country of origin, status of employment, and relationship status. Further questionnaires were used to assess risk factors like maternal mental health problems, stress, personality development, and infant’s development as well as attachment. All questionnaires were provided in German language.

#### Emotional Availability Scale (EAS)

Maternal sensitivity as primary endpoint was measured at all three measurement points using the Subscale Sensitivity of the EAS [16]. The 10-min free-play interaction were conducted in the Mother-Child Facilities. EAS were coded by an independent and reliable rater, blinded for treatment allocation. The rater used a Likert Scale (1 to 7; with 1–3= at risk, 3.5–4.5= some risk, and 5–7= non risk) to score maternal sensitivity as well as three further maternal EAS dimensions (structuring, nonintrusiveness and nonhostility) and two child EAS dimensions (responsiveness to parent and involvement with parent) to describe the mother-child interaction.

#### Mini International Neuropsychiatric Interview (M.I.N.I)

The Mini International Neuropsychiatric Interview (M.I.N.I.) [38] is a structured diagnostic interview which assesses 20 psychiatric disorders by DSM IV and ICD-10 diagnosis. The interview was coded dichotomous (manifestation of mental health problem or not) and administered by trained interviewers at T1 and T3.

#### Parenting Stress Index (PSI)

The German version of the Parenting Stress Index (PSI) [41, 42] is a 48-item questionnaire that was assessed at all three measurement time points. With its 12 subscales, it comprises characteristics of stress in parents and in child. The questionnaire was assessed at all 3 measure times.

#### Edinburgh Postnatal Depression Scale (EPDS)

The Edinburgh Postnatal Depression Scale (EPDS) [43] is a standard 10-item screening instrument measuring symptoms of maternal postpartum depression. It yields a total score. Scores between 10 and 12 indicate a possible postpartum depression and a score of 13 or higher indicates a fairly high likelihood of postpartum depressive symptomatology. The questionnaire was assessed at T1 and T3.

#### Symptom Checklist (SCL-K-9)

The short version of the Symptom Checklist (SCL-K-9) [44] is a 9-item screening questionnaire based on the original SCL-90-R [45]. The SCL-K-9 measures global mental health symptom severity in psychotherapy representing all 9 scales of the long version but does not differentiate between individual types of symptoms. The questionnaire was assessed at T1 and T3.

#### Parental Reflective Questionnaire (PRFQ-1)

The Parental Reflective Functioning Questionnaire (PRFQ-1) [46] is a validated questionnaire which measures the mother’s ability to mentalize in context of the relationship to her child. The PRFQ has 3 subscales, “Certainty about Mental States” (CM), “Pre-Mentalizing” (PM), and “Interest and Curiosity” (IC). The CM scale describes the opacity of mental states and ranges from a tendency of parents to be overly certain about the mental states of their child, to hypermentalizing or to hypomentalizing [46]. The PM scale describes a nonmentalizing state of mind with an inability of the parent to enter the subjective world of the child. High scores on PM often describe malevolent attributions of the child. The IC scale is a key factor of parental reflective functioning. IC captures the interest to think about the reasons of the child’s behavior. Low scores reflect an absence of interest and high scores reflect intrusive hypermentalizing [46]. The questionnaire was assessed at all 3 measure times.

#### Inventory of Personality Organization (IPO-16)

The Inventory of Personality Organization (IPO-16) [44] is a 16-item screening instrument measuring the severity of personality dysfunction. The questionnaire is standardized, and validated, and was conducted at T1 and T3. The IPO-16 yields a total score with a cut-off score > 2.38 assuming the likelihood of a severe structural deficit [44].

#### Parental Bonding Questionnaire (PBQ)

The Parental Bonding Questionnaire (PBQ) [47] is a 25-item screening instrument measuring the quality of parent-infant relationship. A total score ≥ 26 is suggested to indicate some type of bonding disorder and total scores ≥ 40 to indicate severe bonding disorders or maternal rejection [48].

#### ET-6-6-R developmental test

The developmental status is assessed by the German developmental test ET6-6-R [40]. The ET-6-6-R is a standardized test for children aged 6 months to 6 years that measures the developmental status of the child. The ET-6-6-R has 6 dimensions measuring *body motor activity*, *motor activity*, *cognition*, *language skills*, and *socioemotional development*. The test was measured at T3 and T scores were analyzed.

#### Strange Situation Procedure

The child’s attachment was assessed by the Strange Situation Procedure (SSP) [39] at T3. The SSP is a standardized procedure of eight episodes to record the quality of child’s attachment. The aim of the SSP is to activate the child’s attachment system. The test was recorded on video and coded by a certified and independent rater. It subsequently assigned the children to three organized attachment styles (secure (B), insecure-avoidant (A), insecure-resistant (C)) and one disorganized (D) attachment behavior.

### Sample size

A sample size of *N*=40 was planned for this pilot RCT to be recruited within a period of 24 months. Such a sample size would allow to detect moderate effects of *d* = .45 with a power of 80% in an ANCOVA design. Recruitment stopped after 30 months with *n* = 32 mother-infant dyads being included at this time.

### Data analyses

All analyses were conducted in the R (v3.6.1) environment for statistical computing using RStudio (v1.0.136). Data were stored under pseudonyms till the end of the study and were unblinded for treatment intervention after the last participant has completed the study. Only pseudonymous data sets were analyzed. For the main analyses of the primary and secondary outcomes, ANCOVAs were computed with the outcome measured at T3 as the dependent variable and group (PIP vs. CAU) as the between subjects factor and baseline (T1) measurements as the covariate [49]. A significant effect of group or a significant group*covariate interaction in these analyses indicates group differences at T3 after 6 months of intervention while controlling for between subject differences at the baseline measurement.

Due to the large amount of missing data at T3 (only 13 from 32 datasets are available), this procedure likely underestimates the true effects. Therefore intent-to-treat analyses were computed for continuous primary and secondary outcomes using linear mixed effects models (LMMs) for repeated measurements with restricted maximum-likelihood (REML) estimation. LMMs have the advantage of incorporating all available data and thus being less susceptible by missing data, leading to more precise parameter estimates in case of missing data compared to traditional analyses [50]. With regard to the present examinations, a further advantage lies in the fact that all three measurement time-points (if available) could be incorporated in these models at the same time. Thus, these LMMs included fixed effects of time (T1, T2, T3), group (PIP vs CAU), and the time*group interaction and subject-specific random intercepts. LMMs allow for model comparisons to evaluate model fit and the appropriateness of the model terms. Based on model comparisons using the Akaike Information Criterion (AIC) and likelihood-ratio tests, it was found that for *none* of the outcome measures the inclusion of the group predictor was justified by the data. Therefore, all intent-to-treat analyses are based on simpler models that only included time as the single fixed effect and subject-specific random intercepts. A significant main effect of time thus indicates parallel developments in both intervention groups across the measurement time-points. Due to an ongoing discussion on the computation of degrees of freedom in LMMs [51], significance was evaluated based on the computation of 95% confidence intervals around the parameter estimates (see Table 3). Please note again that sample size is small. The results of this pilot RCT are preliminary and should therefore be interpreted with caution. The focus should remain on the descriptive data and the 95% confidence intervals.

Regarding the categorical primary outcome of attachment behavior as measured by the SSP, differences at T3 were examined with Fisher’s exact test, which provides the probability of getting a result as extreme as the observed. Data of the developmental test at T3 were treated as continuous and simple Student’s t-test computed. Significance level for these tests was set at *α* = .05.

## Results

### Feasibility

**Fig. 1:**
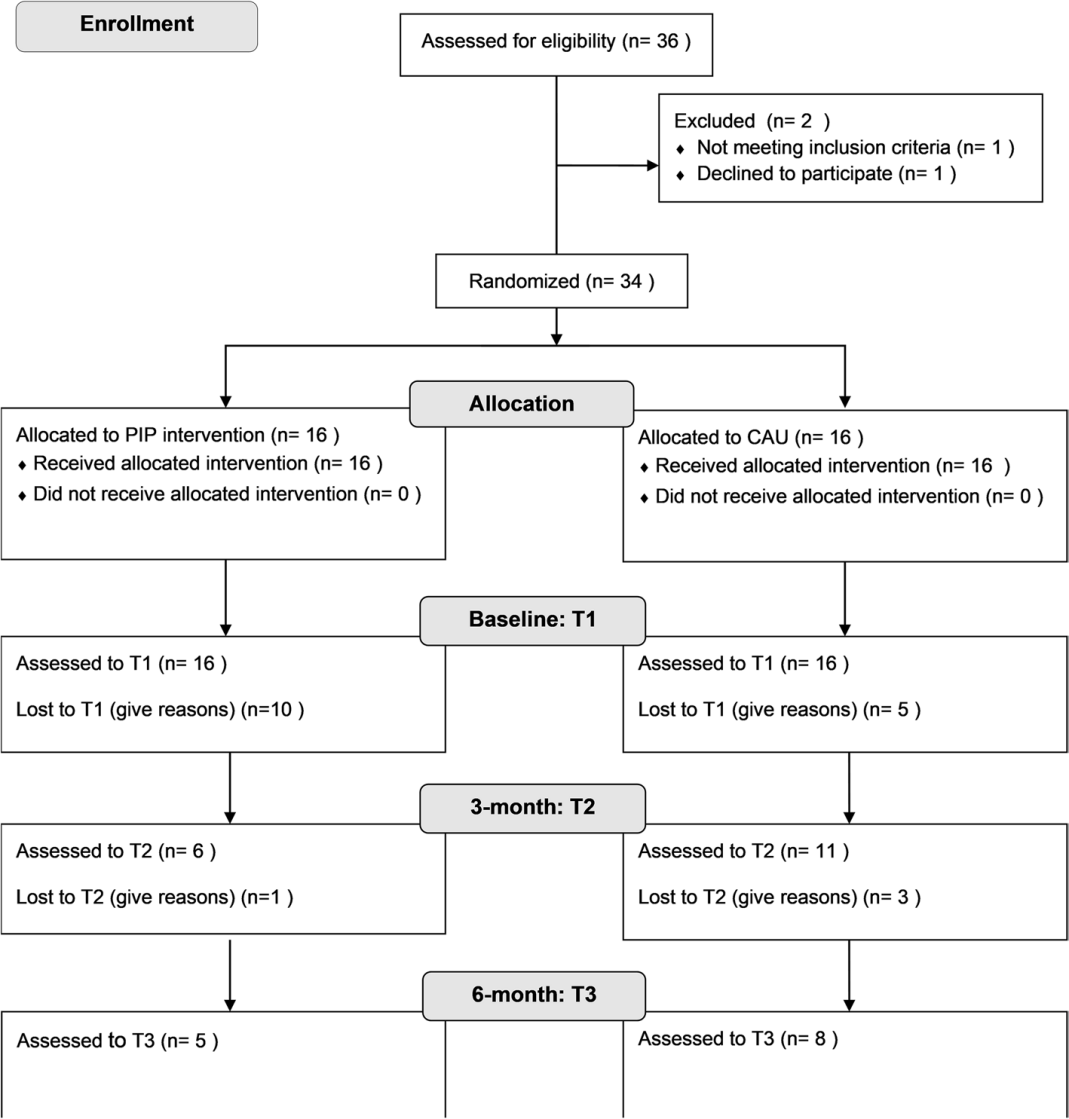
Consolidated Standards of Reporting Trials (CONSORT) diagram describing the participant flow through the study. *PIP* parent-infant psychotherapy, *CAU* care as usual

Motivation to participate and interest in the study were low and retention mostly sustained to ongoing contact and additional in-house support provided by the pedagogical stuff in the facilities. Due to ongoing child-care procedures, mothers were often concerned about participating in the study. There were not enough incentives for remaining in the study or taking part in it in the first place. Eighteen of the 32 dyads (56.3%) dropped out during the course of the study because of emergency removal of the child (*n*=4), they left the facility (*n*=3), the study (*n*=5), or because of other reasons (*n*=6) (see Fig. 1). Two of them were excluded from statistical analysis due to acute psychosis (*n*=1) and drop out before first data collection (*n*=1). There were no significant differences between the dyads which were lost at the 3-month and 6-month assessments in terms of group differences, age, maternal education and employment, marital status, or stress index. This low interest gets also visible by the low numbers of filled out surveys at T3 (see Table 2). None of the self-report surveys were fully filled out or tests conducted by all of the *n* = 14 dyads who remained in the study at T3, leading to as low numbers as *n* = 11 for the SSP analysis or the EAS video recordings. With such a high drop-out rate, feasibility regarding the attrition criterion of 20% could not be successfully determined.

**Table 2:**
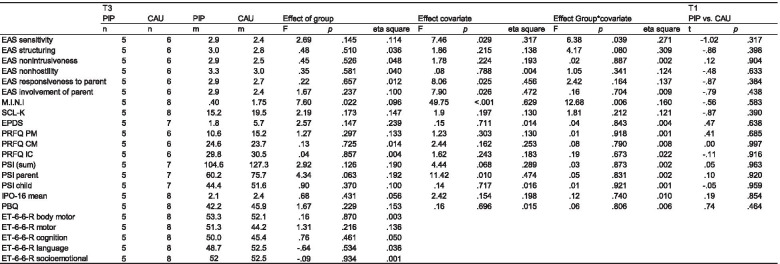
Results of the ANCOVAs for all outcome measures with T3 measurement at 6 months as dependent variable and group (PIP vs. CAU) as the between subjects factor and T1 baseline measurement as covariate

*Abbreviation: EAS* Emotional Availability Scale, *M.I.N.I.* Mini International Neuropsychiatric Interview, *SCL-K* Symptom-Checklist, *EPDS* Edinburgh Postnatal Depression Scale, *PRFQ* Parental Reflective Functioning Questionnaire, *PSI* Parental Stress Index, *IPO-16* Inventory of Personality Organization, *PBQ* Parental Bonding Questionnaire, *ET-6-6-R* Development Test

#### Emotional availability

One participant refused to record videos of her interactions with the child, so only *n*=31 datasets were available for EAS analyses. Overall, it was found that all participants had relatively low scores in the EA-Scales at T1 (*sensitivity* (M = 2.59, SD = 0.62), *structuring* (M = 2.59, SD = 0.60), *nonintrusiveness* (M = 3.00, SD = 0.63), *nonhostility* (M = 3.41, SD = 0.56), *child-responsiveness* (M = 2.67, SD = 0.63), and *child-involvement* (M = 2.20, SD = 0.56)) (see Table 2).

**Fig. 2:**
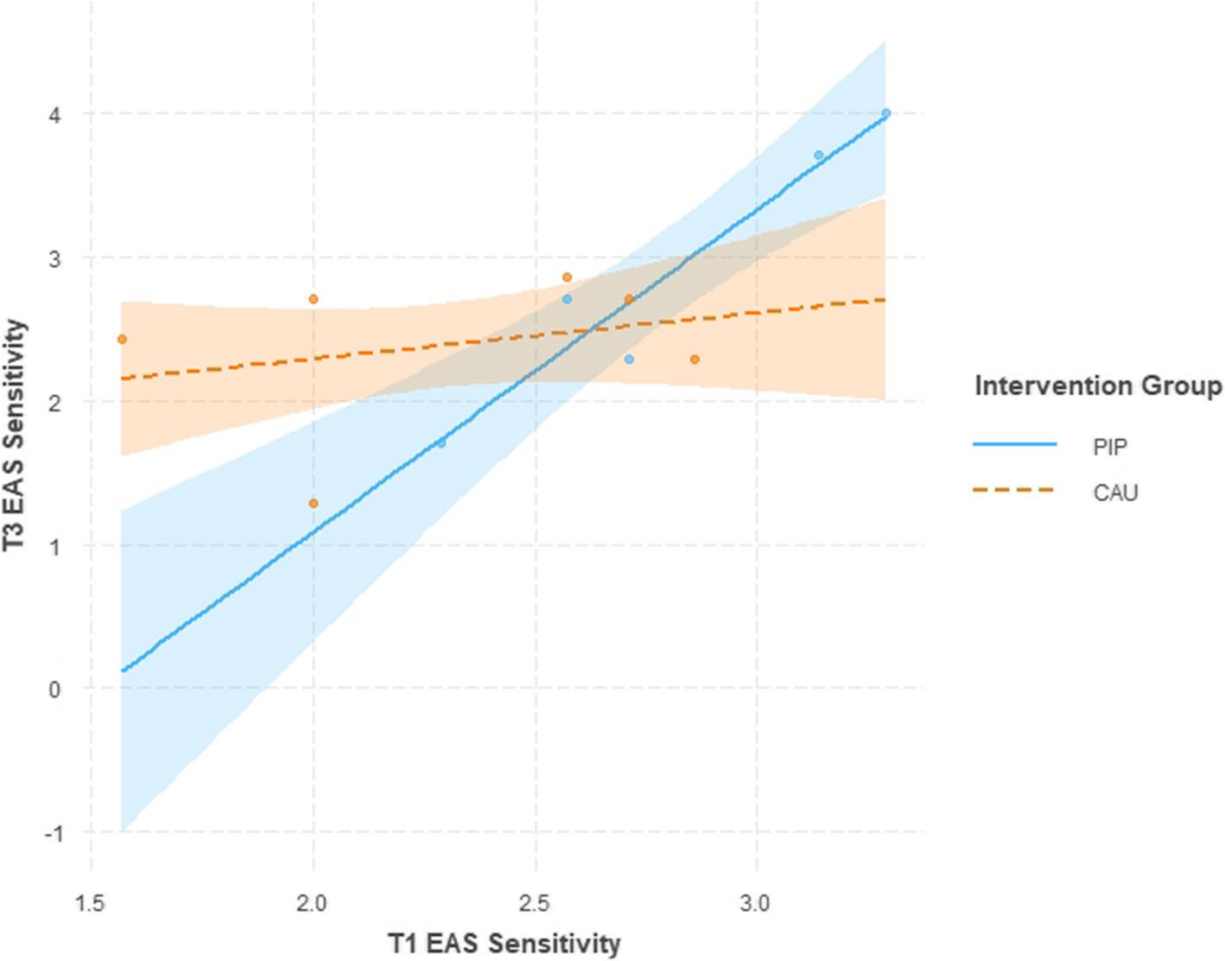
Differential baseline-follow-up relationships in the parent-infant psychotherapy (PIP) and care as usual (CAU) groups in EAS maternal sensitivity

**Fig. 3:**
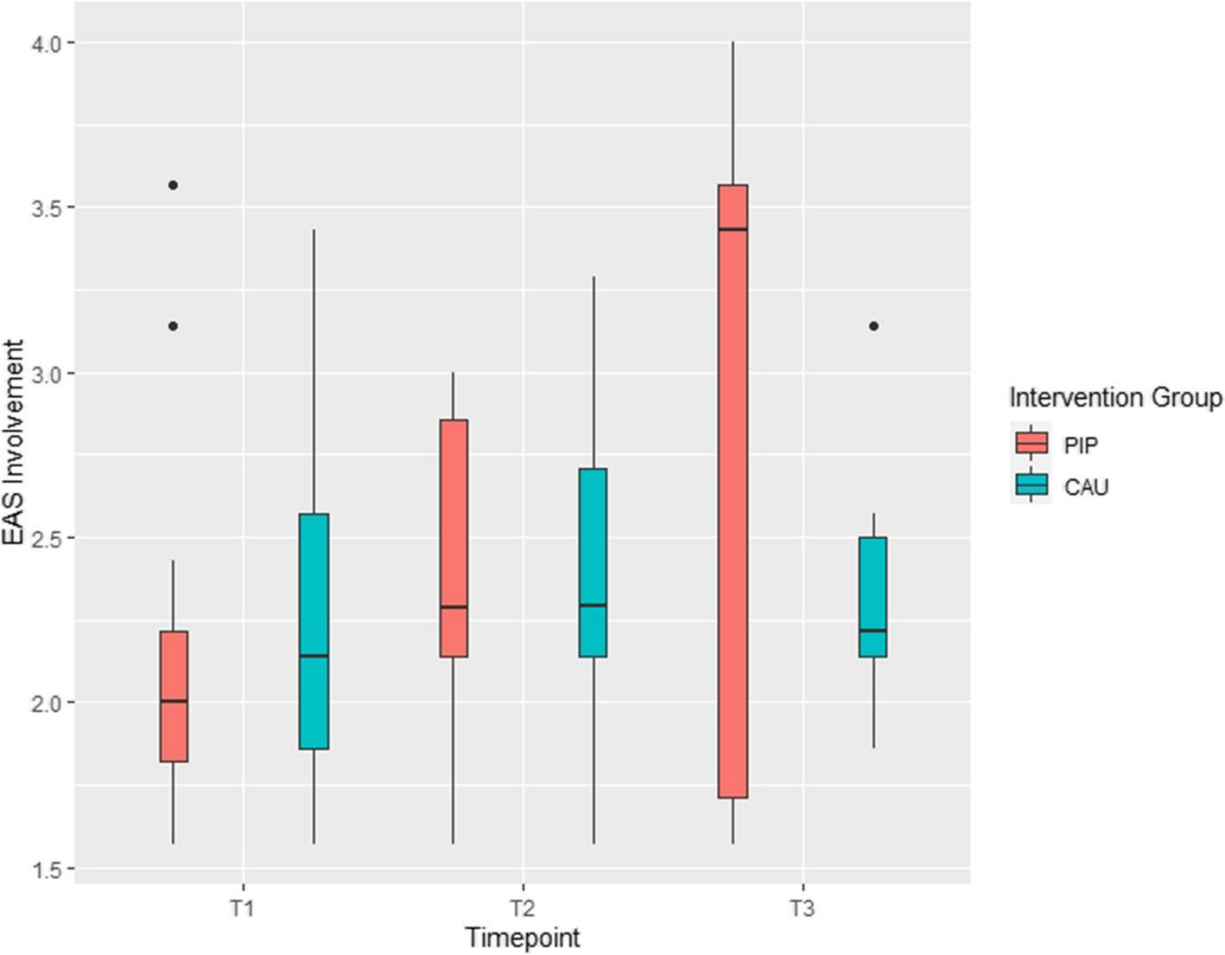
Development of child involvement (EAS) in the parent-infant psychotherapy (PIP) and care as usual (CAU) group across all measurement timepoints. *T1* at baseline, *T2* at 3 months, *T3* at 6 months of intervention

The primary outcome *sensitivity* of the EA-Scales the ANCOVA revealed no effect of group at T3 [F(1, 7) = 2.69; *p* = .145; eta^2^ = .114], but a significant group*covariate interaction [F(1, 7) = 6.38; *p* = .039; eta^2^ = .271] (see Table 2). This effect is due to higher maternal sensitivity at T3 in the PIP group compared to the CAU group moderated by the individual sensitivity score at T1. It seems to indicate a strong relationship between T1 and T3 measures in the PIP group only (see Fig. 2). No effect of time was found in the mixed effects model with all three measurement time-points (both t’s < 1; see Table 3). Thus, PIP intervention had a small moderating effect on EAS sensitivity. No effects incorporating group in the ANCOVA and similar null effects regarding the development across time were obtained for the other EA-Scales (maternal structuring, nonintrusiveness, nonhostility, child’s responsiveness to parent; see Fig. 6), except for “involvement of parent” which increased from T1 to T3 (parameter estimate .34; 95% CI [.01; .66]; see Fig. 3).

**Table 3:** Association between infant’s attachment (SSP) and group (PIP vs. CAU)

	PIP, *n* (%)	CAU, *n* (%)	*n*
Secure	1 (9.1)	1 (9.1)	2 (18.2)
Insecure-avoidant	1 (9.1)	2 (18.2)	3 (27.3)
Insecure-resistant	2 (18.2)	2 (18.2)	4 (36.4)
Disorganized	0 (0.0)	2 (18.2)	2 (18.2)

#### Infant attachment behavior

Results of the child’s primary outcome attachment behavior as measured by SSP classifications after the intervention at 6 months were available from only *n*=11 dyads (PIP *n*=4, CAU *n*=7). In the PIP group, *n*=1 child’s attachment behavior was classified as secure (B), *n*=1 as insecure-avoidant (A), and *n*=2 as insecure-resistant (C). In the CAU group, *n*=2 children’s attachment behavior was classified as secure (B), *n*=1 as insecure-avoidant (A), *n*=2 as insecure-resistant (C), and *n*=2 as disorganized (D). Although there were proportionally more infants in the CAU group who were classified as disorganized, the SSP four-way classifications were not significantly different between groups (*p* = .857, see Table 3).

#### Maternal reflective functioning

No group differences were obtained for the self-reported reflective functioning measured by the PRFQ-1 subscales (all *p-values* >.290, see Table 2). Intention-to-treat analyses incorporating all three time-points revealed that the subscale “Interest and Curiosity” (IC) significantly decreased from T1 to T2 (parameter estimate −2.68; 95% CI [−4.99; −.32]) and similarly from T1 to T3 (parameter estimate −3.36, 95% CI [−6.05; −.65], see Table 2 and Fig. 6). No such significant effects were found for “Pre-Mentalizing” (PM) and “Certainty about Mental States” (CM) subscales.

#### Maternal mental health

Overall number of mental health problems is high in the study sample. Twenty-one out of 32 mothers receive at least one diagnosis according to the M.I.N.I. at baseline (PIP *n*=9; CAU *n*=12). Mental health measured at T3 differed between both groups, with CAU participants exhibiting a significantly higher number of mental health problems on average compared to PIP participants [F(1, 9) = 7.60; *p* = .022; eta^2^ = .096] accompanied by a significant group*covariate interaction [F(1, 9) = 12.68; *p* = .006; eta^2^ = .160]. While this effect of group did not remain significant in the subsequent intention-to-treat LMM analysis, a significant development across time from T1 to T3 was found, with a decreasing average number of mental health problems independent of intervention group (parameter estimate −1.13, 95% CI [−1.95; −.32] (see Fig. 6 and Table 4).

**Table 4:** Results of the linear mixed effects models (LMM) for primary and secondary outcomes

				T2-T1				T3-T1			
	m1	m2	m3	Estimate	CI low	CI high	sign.	Estimate	CI low	CI high	sign.
EAS sensitivity	2.59	2.70	2.61	.05	−.21	.31		.05	−.26	.36	
EAS structuring	2.59	2.75	2.88	.15	−.13	.43		.30	−.02	.63	
EAS nonintrusiveness	3.0	2.92	2.65	−.09	−.43	.25		−.30	−.70	.10	
EAS nonhostility	3.41	3.58	3.16	.14	−.14	.42		−.22	−.55	.10	
EAS responsiveness to parent	2.67	2.66	2.76	−.03	−.34	.27		.12	−.24	.49	
EAS involvement of parent	2.20	2.41	2.58	.17	−.10	.44		.34	.01	.66	*
M.I.N.I	2.28		1.23					−1.13	−1.95	−.32	*
SCL-K	18.31		17.85					−.34	−3.78	3.05	
EPDS	10.09		4.08					−6.01	−9.77	−2.25	*
PRFQ PM	13.19	14.69	13.09	1.42	−1.25	4.11		.73	−2.41	3.82	
PRFQ CM	23.94	25.62	24.09	1.69	−1.22	4.60		.35	−3.03	3.70	
PRFQ IC	33.28	31.00	30.18	−2.68	−4.99	−.32	*	−3.36	−6.05	−.65	*
PSI (sum)	118.8	107.2	117.80	−6.62	−16.62	3.10		−.47	−11.79	10.72	
PSI parent	74.16	63.24	69.25	−6.60	−12.11	−1.22	*	−4.77	−11.02	1.42	
PSI child	44.62	44.00	48.58	−.05	−6.20	6.00		4.20	−2.80	11.14	
IPO-16 mean	2.28		2.42					.03	−.33	.35	
PBQ	22.2		44.5					22.27	12.57	31.98	*

*Abbreviation: EAS* Emotional Availability Scale, *M.I.N.I.* Mini International Neuropsychiatric Interview, *SCL-K* Symptom-Checklist, *EPDS* Edinburgh Postnatal Depression Scale, *PRFQ* Parental Reflective Functioning Questionnaire, *PSI* Parental Stress Index, *IPO-16* Inventory of Personality Organization, *PBQ* Parental Bonding Questionnaire; T1 = baseline, T2 = 3 months, T3 = 6 months; m1, m2, m3 = mean values at T1, T2, T3; CI low, CI high = lower and upper limit of the 95% confidence interval (CI) of the parameter estimate, * = *p*<.05

In comparison, the number of SCL-K-9 symptoms was higher at T3 for CAU compared to PIP (M_PIP_ = 15.2, M_CAU_ = 19.5) but this effect did not reach significance [F(1, 9) = 2.19; *p* = .173; eta^2^ = .143], and there was no development across time (see Table 2).

**Fig. 4:**
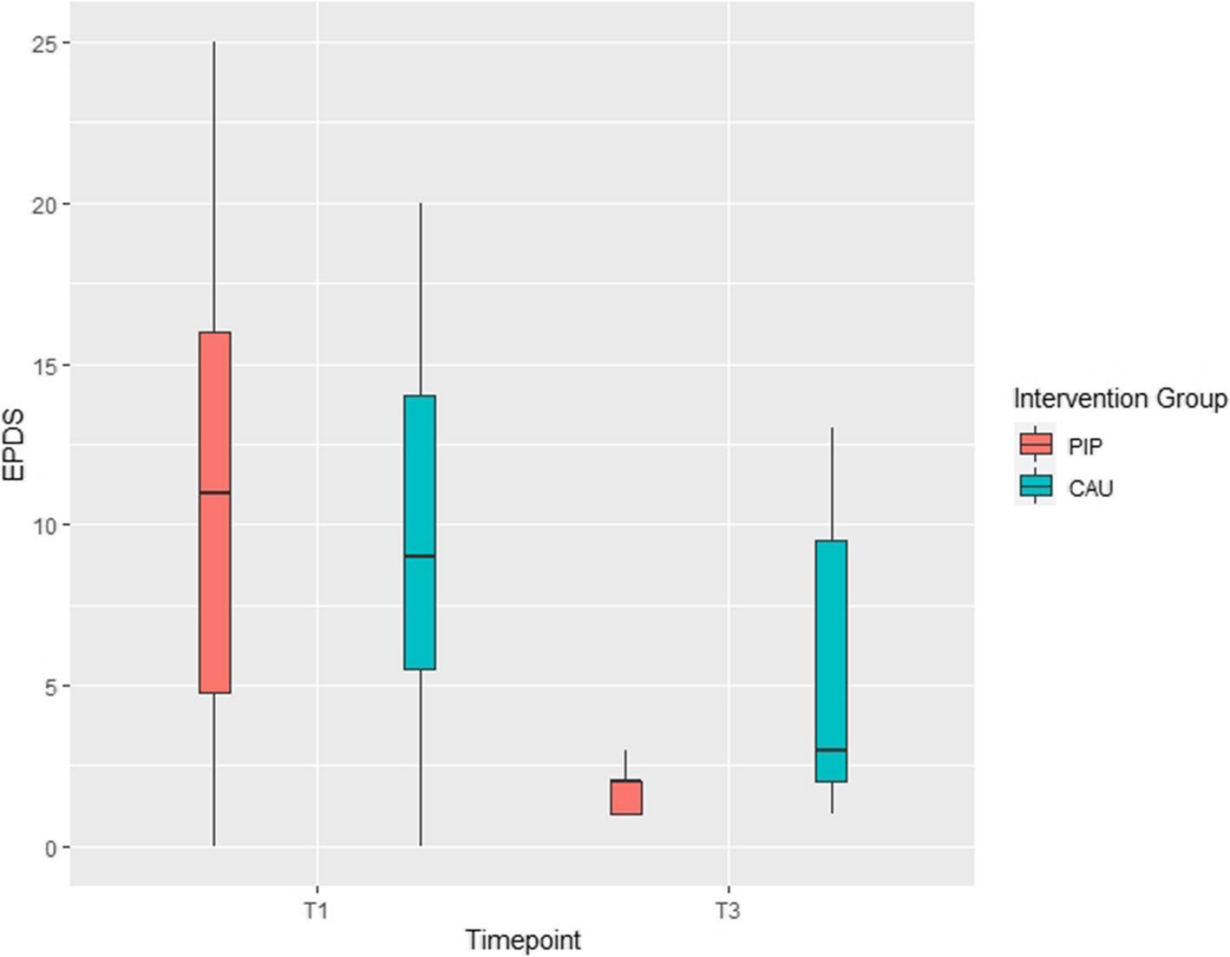
Depressive symptomatology (EPDS) in the parent-infant psychotherapy (PIP) and care as usual (CAU) group at baseline (T1) and 6 months follow-up (T3)

Regarding postpartum depression scores, at T3 descriptive differences were found with higher scores in the CAU group (M_CAU_ = 5.7) compared to PIP group (M_PIP_ = 1.8), a difference that again did not reach significance [F(1, 8) = 2.57; *p* = .147; eta^2^ = .239]. Intention-to-treat analysis revealed that independent of group membership the scores significantly decreased between T1 and T3 (parameter estimate −6.01, 95% CI [−9.77; −2.25], see Table 2 and Fig. 4).

#### Maternal stress

While sum scores of maternal stress reveal no difference between both intervention groups at T3 [F(1, 8) = 2.92; *p* = .126; eta^2^ = .190], the maternal distress subscale was higher at T3 in CAU group (M_CAU_ = 75.7) compared to PIP group (M_PIP_ = 60.2), an effect that approached significance in the ANCOVA [F(1, 8) = 4.34; *p* = .063; eta^2^ = .192]. No such group effect was found in the distress with the child subscale [F(1,8) = .90; *p* = .370; eta^2^ = .100] (see Table 2). In the LMM analysis, independent of group a development for the maternal distress subscale was revealed for the T2-T1 comparison (parameter estimate −6.60, 95% CI [−12.11; −1.22]) with an overall reduction of maternal distress compared to baseline assessment (see Fig. 6). No such effects were obtained for the sum score or the distress with the child subscale or the T3-T1 comparison (see Table 4).

#### Personality organization

The evaluation of the severity of personality dysfunction assessed by the self-report IPO-16 inventory revealed no significant effects (see Table 2)**.**

#### Maternal bonding

**Fig. 5:**
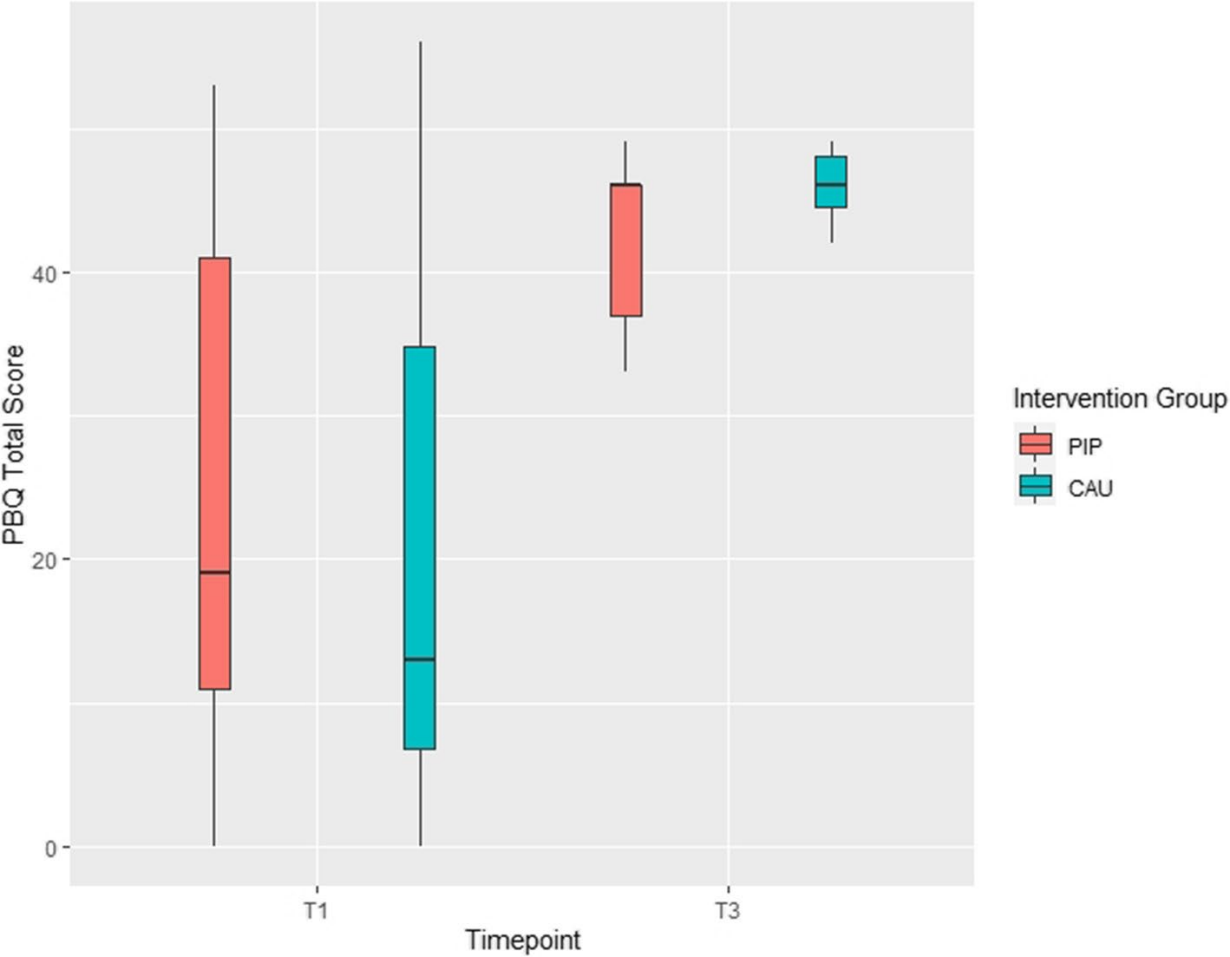
Parental bonding (PBQ) in the parent-infant psychotherapy (PIP) and care as usual (CAU) group at baseline (T1) and 6-month follow-up (T3)

Descriptively, the maternal bonding as assessed by the PBQ at 6 months is at a level that indicates a bonding disorder in most participants (M = 44.5, range 33 to 49) with 9 out of 11 mothers receiving scores above the upper cut-off of a total score of 40. Of interest is that at baseline much lower scores are found: 20 out of 32 mothers scored below 22 (see Fig. 5). This effect of time gets significant in the LMM analysis: independent of group the total scores increase from baseline assessment to 6-month follow-up (parameter estimate 22.27, 95% CI [12.57; 31.98]) which indicates a decline in the self-reported quality of the mother-infant relationship across time. No significant effects involving group were revealed in the ANCOVA (see Table 2).

#### Child development

At T3 only data from 13 children were available (PIP *n*=5; CAU *n*=8). Descriptively, across all five subscales, only children in the CAU group were classified as having a “serious deficit in development” (in 3 out of 5 classifications). Still, the group comparison revealed no significant group differences (all *p*-values > .216, see Table 2).

**Fig. 6:**
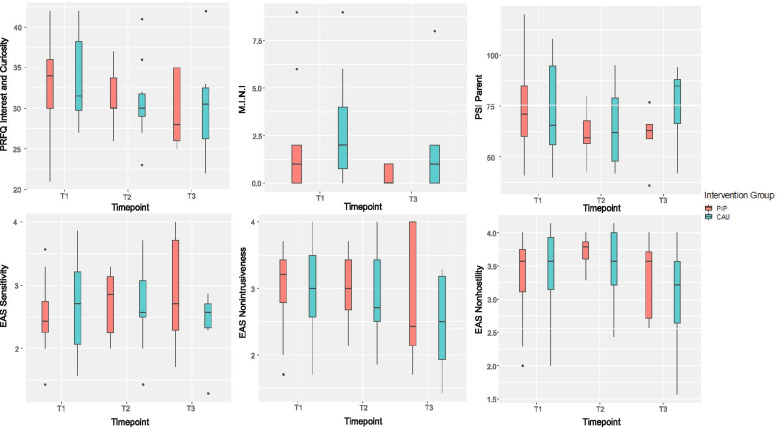
Display of secondary outcomes comparing parent-infant psychotherapy (PIP) and care as usual (CAU) group across measurement time points. *M.I.N.I.* Maternal mental health problems, Interest and Curiosity (PRFQ, parental reflective functioning), parenting stress (PSI), EAS sensitivity, nonintrusiveness and nonhostility, T1 at baseline, T2 at 3 months, and T3 at 6 months

## Discussion

The randomized pilot trial examined feasibility of recruitment and potential efficacy of PIP as an additional offer besides the social and pedagogical care in German Mother-Child Facilities, with a particular focus on maternal sensitivity and child attachment in a high-risk population. It was found that PIP had small but consistent effects on maternal sensitivity but no effects on children’s attachment behaviour. The results also reveal that maternal mental health, stress, and reflective functioning decreased, with some evidence of group differences. However, overall low sample size after attrition lowers the generalizability and interpretations of the present results.

Mothers in the PIP group at 6 months reported a lower number of mental health problems compared to CAU participants. Independent of group all mothers had a lower number of mental health diagnoses after 6 months of living in Mother-Child Facilities. In the same direction and with large effect sizes (but not significant), mothers in the PIP group had fewer depressive symptoms and lower distress and showed increased sensitivity towards the child after 6 months of intervention. The effect in the primary outcome maternal sensitivity was only found as a significant interaction: In CAU participants, sensitivity at baseline has no relationship with post-intervention sensitivity scores, while in the PIP group mothers with higher baseline levels benefitted most from PIP intervention. According to the meta-analysis of Barlow et al. [33], PIP is more effective in high-risk populations, and the present data in sum fit into this notion. It is still puzzling, why mothers with already higher baseline levels benefited more. It could be hypothesized that higher sensitivity levels at the beginning and thus a better understanding of the child’s needs and signals are an eligible ramp for further improvement, but additional experimental data is needed, due to the overall small sample size and the high attrition rates at T3.

At baseline, all of the examined mothers show relatively low levels of emotional availability, particularly regarding maternal sensitivity. Sensitivity is discussed as one of the key factors for the child’s healthy development. The present results reveal that these mothers at high-risk predominantly show difficulties in reading the emotional signals of their infants and their infants tend to show a poorer development. Whereas limited maternal emotional availability is seen a predictor of insecure attachment [17], one might hypothesize that a lack of sensitivity in adulthood is related to an immature ability to recognize or process infant’s emotional states or impeding sensitive parenting [52]. Studies have shown that especially younger mothers have problems in understanding the child’s needs adequately and tend to misunderstand the infant’s level of development, giving them an inappropriate meaning or show more instrumental (and less affectionate) behavior [6, 53]. Children in the present study tend to show more passive behavior with less involvement and responsiveness in the interaction, which could be a consequence of maternal insensitivity and a factor for developmental delays. The overall low EA-Scores at baseline and 6 months indicate an increased risk of transmissions of rearing experiences and malignant patterns to the offspring in the at high-risk study sample. All these findings can also ultimately lead to an insecure attachment development [5, 6]. It could be hypothesized that these results may be due to the cumulative risk factors of having an own history of harsh life experiences, trauma, or missing positive parenting experiences, leading to maternal insensitivity in the interaction with the child [6, 17].

As seen from EA-Scales and SSP, all participants were at high risk of disturbance in early mother and child interaction at baseline and, respectively, regarding attachment development at 6 months. The majority of the infants are insecurely attached to their mothers and thus could be expected to have more difficult developmental trajectories [4, 17]. These findings from a high-risk population are in contrast to the results of Barlow et al. [33] showing that parents who received PIP were more likely to have an infant who was securely emotionally attached after intervention. Due to the missing premeasurement of maternal attachment, the present findings must be interpreted with caution, although, it is well known that maternal attachment style is related to the style they give to their own offspring [53]. Recent research has revealed a link between attachment classifications among mothers with a history of childhood abuse and neglect as well as insecure and disorganized attachment classification [54, 55]. A similar difficult prognosis can be drawn from the postpartum bonding results which indicate difficult to severe bonding deficits in all mothers at 6 months. Here, in contrast to maternal sensitivity, no differences between both groups were found. Thus, neither care in Mother-Child Facilities nor 6 months of PIP intervention had a positive effect on the maternal bonding—despite the effects on maternal sensitivity and reflective functioning. Overall, the PBQ results seem at odd with the literature. A deficit in bonding cannot be predicted from previous studies which mainly agree in that social care and psychotherapeutic intervention increase maternal bonding processes, i.e., positive feelings, and affection towards the child (e.g., [47]). Though case reports exist where bonding difficulties last beyond the end of treated postpartum depression [56], also there are methodological problems associated with the use of PBQ in a high-risk sample. With very low scores at baseline, it seems similarly likely that mothers reported socially desired behavior. After 6 months of care and with improved reflective functioning and the capacity to mentalize, a better description of their bonding can be found. Such tendencies should be addressed in future research and compared with further samples of mothers.

The present analyses reveal that mothers with higher levels of sensitivity benefit most from PIP in this high-risk sample and are more likely to have a better relationship to the child. Thus, there appears a clear impact on mother’s well-being. In support of this first evaluation, it was also found that *interest and curiosity* in the child’s behavior, a factor of parental reflective functioning, decreased in both intervention groups. This can be either a result of an improved understanding of the child and its signals [6] or due to the child’s increasing age and its ability to better communicate with the parent. Nevertheless, in comparison with the high (hypermentalizing) scores at baseline, a lower interest and curiosity score in the child’s mental state might be interpreted as a better ability to be less intrusive and to mentalize with regard to the child and in turn have a more responsive child. It has also to be noted that *certainty about mental states* and *interest and curiosity* still remain with striking high scores during the 6 months, indicating an overall low ability for maternal reflective functioning and mentalizing in context of the child [57]. The overall level of maternal reflective functioning did not increase significantly over time and is still below the normal levels of reflective functioning and mentalizing (cf. [57]), replicating findings from high-risk groups of imprisoned mothers [58].

Parenting distress was reduced in the first 3 months, an effect that was not found at 6 months anymore. It seems that living in the Mother-Child Facilities initially lowers maternal stress levels in both groups. But with longer stay, one could hypothesize distress levels increase again. This might be related to the support received in these facilities and in the study, which initially leads to emotional well-being of the mother [59]. One might further hypothesize that the care provided in these special homes and the influence of other peers initially has a positive impact on the mother’s emotional well-being. At first, living in the mother child homes with 24/7 care may improve maternal confidence and reduce feelings of isolation [27]. The support offered by the facilities might bring relief from acute problems in the first months. With longer stays, the everyday life becomes however increasingly stressful; potential child welfare or child-care proceedings and the question of the dyad’s separation might interfere with maternal stress level. Also living together with similarly burdened (adolescent) mothers can be experienced as stressful. The multi-professional team, which was first seen as supportive, can change into a burden task. However, these results have to be interpreted with caution, because the current study did not measure the subjective experiences of the participants.

These results also signal that all dyads in the sample were at an increasing risk for child maltreatment. The mixed model analyses reveal some evidence that living in Mother-Child Facilities, with its social and pedagogical care, improves maternal mental health symptoms and functioning. Findings that are in line with the meta-analysis of Taubner et al. [32] who reveal that early intervention programs (without therapeutic elements) lead to symptom reductions and can be regarded as true program effects. It also has to be noted that the majority of the mothers show mental health problems without getting adequate therapeutic support. Across measurement points, depressive symptomatology and the number of mental health problems decreased and involvement of the mother with the child increased. Thus, independent of intervention group, the risk to the child’s welfare was lowered. Again, the missing PIP versus CAU group effects seems at least partly be attributable to overall low sample size and high drop-out rates. Still, the results point to slight group differences at 6 months, in that only in the group with additional psychotherapeutic intervention the distress level at 6 months period remains at a lower level. In line with the aims of PIP, one might assume that mothers in the PIP group are more sensitive with the child’s signals and thus start to interpret signals differently leading to lower levels of reported distress. With regard to child’s development, only the children in the CAU group were at higher developmental risk after 6 months, but without a significant group difference. It seems likely that the decrease of maternal distress may provide a resilience factor for the infant’s development.

Given the small PIP effects, it has to be discussed why the results in sum did not mirror previous reported findings and the initial hypotheses. It seems likely that maternal painful and traumatic experiences may be limiting the capacity to reflect thoughts, feelings, and intentions of the child [59, 60] and undergo therapy outcome. Hence, this very difficult sample of predominantly young mothers shows low motivation of being treated. While positive therapy outcome is associated with the participants motivation and the willing to change [28], poor therapy outcome is also related to personality organization and the structural level of mothers. Koelen et al. [61] highlight in a systematic review the personality organization as a predictive factor for success in therapy. According to this, low structural levels of personality organization can impede therapy outcomes, findings which seem related to the present findings. Nearly half of the participants show at baseline a personality organization that can be interpret as indicating a structural deficit. Hence, parents with difficult and ongoing mental health issues are having the worst therapeutic outcome [62]. The uncertainty about what PIP entailed could be another reason for poor treatment outcome and high drop-out rates [63]. Ransley et al. [28] further highlighted that treatment expectations and the ability to speak about own concerns not to speak about past (traumatic) experiences in therapy sessions are, among others, key factors for enhancing parental reflective functioning in therapy.

### Limitations

Although there is small but consistent evidence for the effectiveness of PIP, there are also limitations including high drop-out rates, the selection of questionnaires, and the follow-up period. It has to be noted that the results should be interpreted with caution as the heterogenous and small sample size could limit the generalizing of results. Small sample sizes are likely to have contributed to a lack of statistical power to detect significant effects. The current findings reveal several large effects (in terms of effect sizes eta^2^ > .125) after 6 months that are reported as not significant. A lack of a long-term follow-up and a larger sample size could have brought up more stable results, i.e., mothers’ improvements seen in the measures had not yet been consolidated enough to be internalized and reflected in the mother’s outcomes and child’s secure attachment development. A long-term follow-up is required to understand the sustainability of treatment effects.

The high drop-out rates seem related to the high-risk population assessed. Many of these mothers are concerned discussing their past (traumatic) experiences in therapy sessions. Treatment barriers are often one of the main explanations for early drop out [64]; hence, demography and own expectations are discussed as having the biggest impact [28]. In line with previous studies [32], it was observed that this at-risk population had only little therapeutic motivation or intention of being treated, although there is an obvious need for treatment. In many cases, the burdened mothers are unable to guarantee appointments and continuity [32]. The anecdotal qualitative evidence reported by the study staff leads to a picture where it is more difficult for the researchers and the psychotherapists to establish a positive working relationship to the mother. Thus, new models and methods to establish working alliances and a therapeutic relationship should be incorporated in future adaptations of PIP for these at-risk populations, with a particular focus on the needs of these mothers and children. For example, PIP interventions could also be offered at home and with a flexible frequency. Grosse Holtforth et al. [65] argued that frequency, regularity, and flexibility are key factors for the therapeutic relationship, factors which are difficult to establish for this risk sample. Most of these mothers never experienced stable relationships. Thus, a trustful working alliance needs more time and effort to be developed in these settings [63].

Further limitations include the recorded 10-min mother-child play interactions for EA coding which may be insufficient to capture the overall quality of mother-infant relationship and may limit the external validity of the measure. Standard recommendations are 20-min intervals although meaningful results with shorter sessions have also been reported [66]. The reliance on screening questionnaires like IPO-16 and SCL-K and the self-report measurements can also lead to distortions and bias of results. Although the study focused on mother and child, more instruments should have been used to assess the development status and outcomes of the child. The child could be an important indicator for the mother-child relationship, but only the child’s developmental progress and attachment is assessed in the present study. Child symptomatology and mental health could be added in future research. On the other hand, in terms of feasibility one might wonder whether the number of self-report measures could have contributed to the high drop-out rates in the present study. Overall motivation to participate, interest, and understanding of empirical research was found to be low in the sample. Future studies should aim to further reduce the number of questionnaires in exchange for more external and objective evaluations by nurses and therapists. The results of the feasibility study partly contributed to the development of the RCT design of two large-scale PIP intervention studies [67, 68]. The missing psychiatric diagnoses of mothers as inclusion criteria likely also affect therapy outcome. Finally, it must be noted that there are many other risk factors which were not assessed in the present study, but which may contribute to disturbances in mother-child dyad in the Mother-Child Facilities. It is a combination of cumulative factors leading to the high risk of child welfare, abuse, and adverse mother-child development. Potential child-care proceedings, transition from adolescence to adulthood, problems with peers, fathers, or social workers might interfere with treatment outcomes, enhancing the maternal stress level and interfere with a secure attachment establishment.

Nevertheless, improvements in the whole sample due to the care provided in these Mother-Child Facilities might lower the likelihood of finding effects in the PIP intervention group. More effort should be taken to evaluate the effects of the social and pedagogical care in the Mother-Child Facilities with a particular focus on psychological effects like parenting stress and reflective functioning and also mental health. Thus, besides the necessity of larger samples sizes, more or different control groups are demanded in future studies of these at-risk samples.

## Conclusion

This pilot study established a first randomized controlled trial with mother-infant dyads at high-risk living in German Mother-Child Facilities, with a particular focus on the impact of additionally offered PIP on the mother-infant-relationship. Due to the high drop-out rate, only preliminary conclusions can be made at this stage. Some evidence was found that all mothers benefit from the social and pedagogical support offered by the staff in these facilities. Group differences were small and are best documented for reduced mental health problems in the psychotherapy intervention group with some evidence of additional improvements in maternal sensitivity.

In order to prevent a transgenerational transmission of mental health problems and malignant attachment patterns, it is necessary to continue the evaluation of such psychotherapeutic interventions that target psychologically and socially burdened mothers (and fathers) of infants in Mother-Child Facilities. It is alarming, that most of the mothers assessed show mental health problems, and depressive symptomatology and have no psychotherapeutic support offered. Considering the impact of maternal pathology on the child’s development, it is crucially important to establish further research with psychotherapeutic interventions.

Further research is needed to evaluate the effectiveness and need of PIP in Mother-Child Facilities, but also to evaluate the effectiveness of the preventive and social care. The next step of this project is to design a larger RCT by thoroughly considering the problems with recruitment and retention. Before conducting a RCT qualitative studies would be beneficial. Emphasis should be given on the motivational side, for example by providing more information on the positive effects such interventions have for the participating dyads. Retention might be increased by the availability of home visits in case a mother-child dyad leaves the facility. Such a future study needs also to consider a potential effect of social and pedagogical care. The present results show, however, that maternal emotional availability can be improved, and both interventions support the at-risk mothers to gain a better understanding of their children and their needs. With its psychodynamic, mentalization-based approach, PIP seems to be an appropriate additional offer for these high-risk mothers. However, further research is needed to particularly evaluate the factors and procedural characteristics of an effective intervention in such a group of mother-infant dyads living at-risk. It still remains unclear which change mechanisms underlie the effects of socio-pedagogical support and to what extent they are beneficial for this specific risk population or transferrable to other samples.

In sum, the findings support the notion that this first empirical evaluation of PIP in Mother-Child Facilities and with mothers at high-risk reveals a positive impact on the dyad. It clearly has to be noted that this high-risk sample is difficult to assess. From great clinical importance is the question of what works for whom and what enables (adolescent) mothers to engage (or not) in study flow and therapy. A further feasibility result seems to be the understanding that it might be more important to first fulfill basic needs such as shelter and safety, before embarking on a psychotherapeutic intervention. It seems likely that PIP is more effective once the environment had stabilized for the mother-child dyads—which should be addressed in future research. In order to capture this particular group of mothers and their children at high-risk properly, research is needed considering the participants’ subjective world as well as effects of psychotherapies and long-term consequences.

## Data Availability

The datasets used and analyzed during the current study are available from the corresponding author on reasonable request.

## Abbreviations

RCT: Randomized controlled trial
PIP: Parent-infant psychotherapy
CAU: Care as usual
EAS: Emotional Availability Scale
M.I.N.I: Mini International Neuropsychiatric Interview
PSI: Parenting Stress Index
EPDS: Edinburgh Postnatal Depression Scale
SCL-K-9: Symptom Checklist
PRFQ-1: Parental Reflective Questionnaire
IPO-16: Inventory of Personality Organization
PBQ: Parental Bonding Questionnaire
ET-6-6-R: Developmental test
SSP: Strange Situation Procedure
LMMs: Linear mixed effects models
REML: Restricted maximum-likelihood
